# Artificial Intelligence Applications in Health Care Practice: Scoping Review

**DOI:** 10.2196/40238

**Published:** 2022-10-05

**Authors:** Malvika Sharma, Carl Savage, Monika Nair, Ingrid Larsson, Petra Svedberg, Jens M Nygren

**Affiliations:** 1 Department of Learning, Informatics, Management and Ethics Karolinska Institutet Medical Management Centre Stockholm Sweden; 2 School of Health and Welfare Halmstad University Halmstad Sweden

**Keywords:** artificial intelligence, health care, implementation, scoping review, technology adoption

## Abstract

**Background:**

Artificial intelligence (AI) is often heralded as a potential disruptor that will transform the practice of medicine. The amount of data collected and available in health care, coupled with advances in computational power, has contributed to advances in AI and an exponential growth of publications. However, the development of AI applications does not guarantee their adoption into routine practice. There is a risk that despite the resources invested, benefits for patients, staff, and society will not be realized if AI implementation is not better understood.

**Objective:**

The aim of this study was to explore how the implementation of AI in health care practice has been described and researched in the literature by answering 3 questions: What are the characteristics of research on implementation of AI in practice? What types and applications of AI systems are described? What characteristics of the implementation process for AI systems are discernible?

**Methods:**

A scoping review was conducted of MEDLINE (PubMed), Scopus, Web of Science, CINAHL, and PsycINFO databases to identify empirical studies of AI implementation in health care since 2011, in addition to snowball sampling of selected reference lists. Using Rayyan software, we screened titles and abstracts and selected full-text articles. Data from the included articles were charted and summarized.

**Results:**

Of the 9218 records retrieved, 45 (0.49%) articles were included. The articles cover diverse clinical settings and disciplines; most (32/45, 71%) were published recently, were from high-income countries (33/45, 73%), and were intended for care providers (25/45, 56%). AI systems are predominantly intended for clinical care, particularly clinical care pertaining to patient-provider encounters. More than half (24/45, 53%) possess no action autonomy but rather support human decision-making. The focus of most research was on establishing the effectiveness of interventions (16/45, 35%) or related to technical and computational aspects of AI systems (11/45, 24%). Focus on the specifics of implementation processes does not yet seem to be a priority in research, and the use of frameworks to guide implementation is rare.

**Conclusions:**

Our current empirical knowledge derives from implementations of AI systems with low action autonomy and approaches common to implementations of other types of information systems. To develop a specific and empirically based implementation framework, further research is needed on the more disruptive types of AI systems being implemented in routine care and on aspects unique to AI implementation in health care, such as building trust, addressing transparency issues, developing explainable and interpretable solutions, and addressing ethical concerns around privacy and data protection.

## Introduction

Artificial intelligence (AI) is often heralded as a potential disruptor that will transform the practice of medicine [1,2]. The promise of AI lies in its ability to process and learn from large volumes of data and capture patterns otherwise difficult for humans to identify. This ability has raised questions and worries about liability and risks, in particular related to the level of autonomy granted to AI applications [3]. Others see a role complementary to humans; for example, decision support or decision augmentation where humans (in the roles of clinicians or programmers) provide oversight and collaborate [4-7]. The latter approach has been demonstrated to yield superior performance compared with experts alone [8]. Other benefits include improved patient outcomes, error reduction, health system optimization, cost reductions, and increased value [6].

The amount of data collected and available in health care, coupled with advances in computational power, has contributed to advances in AI applications [9] and an exponential growth of publications on AI in health care, with >10,000 records on PubMed in 2021 alone. Included in this are multiple reviews across medical specialties that explore the potential roles of AI to augment health care delivery [10-14]. These include diagnostic (eg, early cancer diagnosis, diabetes retinopathy screening, or COVID-19 diagnosis based on computed tomography images), therapeutic (eg, precision medicine in chemotherapy and for combination drug therapy), and regulatory or administrative applications (eg, coding of records or economic evaluations), as well as for population health management (eg, public health surveillance or predictive epidemiological modeling) [15-21].

However, the development of AI applications does not guarantee their adoption into routine health care practice. Research has identified a number of factors influencing adoption of innovations. These include context (eg, economic and political context, laws and regulations, and sociocultural factors), organization (eg, organizational structure, resources, and processes), group (eg, professional values and cultures), individual (eg, attitudes, motivation, user satisfaction, and trust), and technology (eg, usability, design, accuracy, and explainability) [22,23]. This suggests a need to know more about how AI can be implemented in health care, not only as an innovation but also with respect to its unique potential and associated concerns.

Previous reviews have tended to focus only on some aspects of the process of implementation of AI in health care; for example, regulation and legal issues [24,25], trust and ethics [24-29], clinical and patient outcomes [30-32], and economic impact [33]. Others have focused their studies on specific AI applications for health care, such as predictive medicine, diagnostics, and clinical decision-making [9,30,34,35]. A few reviews have been more overarching, focusing on coproduction processes [36], implementation frameworks [37], and critical implementation barriers or success factors [38] that could inform the development of relevant implementation strategies of AI technology. Generally, it is argued that the implementation of AI in health care could significantly improve patient and health care outcomes, but none of these reviews have actually explored the knowledge base of real-world implementation in everyday clinical practice.

Given the resources invested in developing AI applications and the risk of reproducing already investigated aspects of effective AI applications to support, augment, and perhaps even transform health care for patients, staff, and society, we sought to explore how the implementation of AI in health care practice has been empirically investigated in the research literature.

## Methods

### Study Design

**Figure 1:**
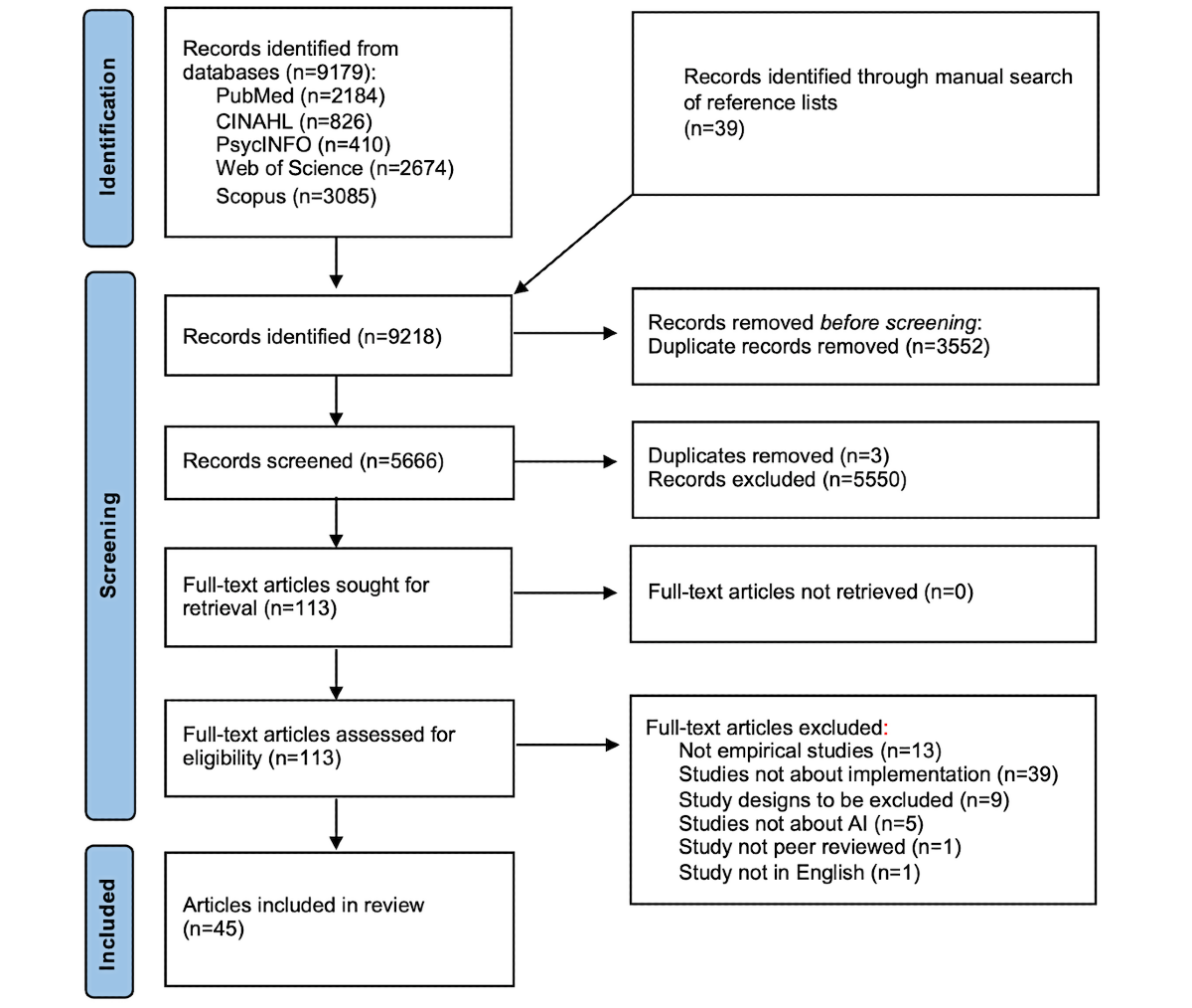
PRISMA (Preferred Reporting Items for Systematic Reviews and Meta-Analyses) flowchart. AI: artificial intelligence.

We chose a scoping review methodology in line with the Arksey and O’Malley framework [39] and reported according to the PRISMA-ScR (Preferred Reporting Items for Systematic Reviews and Meta-Analyses extension for Scoping Reviews) checklist (Figure 1) [40]. A previous review suggested that implementation of AI in health care was not well studied [37]. A scoping review would thus enable a mapping of the “extent, range and nature of research activity” in this emerging area of research [39].

### Identifying the Research Question

To address our aim, we formulated three research questions:

1. What are the characteristics of research on implementation of AI in practice?
2. What types and applications of AI systems are described?
3. What characteristics of the implementation process for AI systems are discernible?

### Identifying Relevant Studies

We focused our search, with support from a university librarian, by iteratively testing synonyms for 3 concepts: *artificial intelligence*, *health care*, and *implementation* (Textbox 1). For the purposes of clarity, we differentiated between AI algorithms and models (the actual code), AI applications (the innovation *package*), and AI systems (the application in its context) and used standardized Medical Subject Headings terms and subject headings describing AI and its subcategories provided by the databases used for our searches [41]. Implementation was defined as “An intentional effort designed to change or adapt or uptake interventions into routines,” based on a review of frameworks for the translation of AI into health care practice [37]. Synonyms were joined by the Boolean operator *OR*; next, we combined the search strings for each concept with the Boolean operator *AND* (Multimedia Appendix 1).

To cover content in both general and health- and health care–specific sources, 5 electronic databases were searched: MEDLINE (PubMed), Scopus, Web of Science, CINAHL, and PsycINFO. In addition, we used snowball sampling by manually reviewing reference lists of the review articles we had identified during the screening that might contain relevant references given the topic of the review.

**Concept areas and synonyms used to develop the search strategy.**

**Search concepts, combined using “AND”**

- Artificial Intelligence
- Healthcare
- Implementation

**Search terms, combined using “OR”**

- Artificial intelligence, Neural networks, Deep learning, Machine learning
- Delivery of healthcare, Health care, Healthcare
- Implementation, Improvement, Innovation, Intervention

### Eligibility Criteria

We included peer-reviewed empirical studies published in English between December 2011 and February 2022 because preliminary searches suggested that AI applications in health care are a more recent phenomenon (Table 1).

**Table 1:** Eligibility criteria and their rationale.

Eligibility criteria and variable		Rationale
**Inclusion criteria**		
	Peer reviewed	Greater credibility because the papers have been reviewed by peer experts in the field
	Empirical study design	Empirical studies improve the ability to answer the research questions compared with conceptual commentaries or viewpoints
	Published between December 2011 and February 2022	Given the rapid pace of development of technology and changing data sets, solutions developed before the last decade are likely to be obsolete
	English language	Practical consideration, given the investigators’ language proficiency
**Exclusion criteria**		
	Nonempirical designs, including editorials, commentaries, opinion articles, and reports	Empirical studies improve the ability to answer the research questions compared with conceptual commentaries or viewpoints
	Proof-of-concept, feasibility, or validation studies not related to implementation of artificial intelligence technologies	As the aim was to explore implementation in practice, studies that stop short of that, for example, proof-of-concept, validity, or feasibility studies, should be excluded

### Study Selection

All identified records were imported into the open-access software Rayyan. Duplicates were removed, and the titles and abstracts of the remaining records were screened for eligibility by at least one of the authors. Any uncertainty or conflict was discussed at regular check-ins until consensus was reached among all authors. These discussions were informed by the multidisciplinary backgrounds of the authors. We also continually reviewed our interpretations of the screening criteria, and when questions were raised, we backtracked to ensure that the criteria had been applied correctly and in a universal fashion, independent of who had screened the records. We used the AI screening and highlighting function of Rayyan, but we still screened each record. We also erred on the side of inclusion. Full-text articles were then screened independently by at least two researchers. Conflicts and uncertainty were again resolved through discussion until consensus was reached among all researchers. As we followed the original framework, a quality appraisal of the included studies was not conducted.

### Charting the Data

We developed a data extraction template to chart data for each of the research questions. To define these conceptual areas, we adopted the World Health Organization’s guidance on ethics and governance of AI for health definition of AI (based on a recommendation of the Council on Artificial Intelligence of the Organisation for Economic Co-operation and Development states) [42,43]: “An AI system is a machine-based system that can, for a given set of human-defined objectives, make predictions, recommendations, or decisions influencing real or virtual environments. AI systems are designed to operate with varying levels of autonomy” [42].

The following data were extracted:

1. General information: authors, publication year, country, clinical setting, study aim, and study design
2. Types and applications of AI: AI technology used, type of AI model, type of task performed by AI, level of action autonomy, intended use of AI, and intended user of AI
3. Implementation process: research focus, motives for implementation, elements in the implementation process, and frameworks used

### Collating, Summarizing, and Reporting the Results

The extracted data relating to research questions 1 and 2 were mapped and summarized. A qualitative thematic analysis [44] was used to analyze data associated with research question 3 to summarize the motives for implementation and elements in the implementation process. Articles were read and reread, with initial ideas sorted into either the domain *Motives* behind the implementation or *Elements* in the implementation process. Next, initial codes were identified in each article. The codes were compared based on similarities and differences and collated into potential themes, which were then compared to generate a thematic map that was used to generate clear definitions and names for each theme in the respective domains. Coding and data analysis were performed in pairs, and any uncertainties were discussed among all authors until consensus was achieved.

## Results

### Search Results

We identified 9218 records, of which 9179 (99.58%) were identified through database searches and 39 (0.04%) through a snowball search of reference lists in the review articles (n=36). Of the 9218 records, after removal of duplicates, 5666 (61.47%) records remained, and we screened titles and abstracts. In this screening, 98% (5553/5666) of the records were excluded, and the remaining 2% (113/5666) were assessed for eligibility through full-text review. Of these 113 articles, 68 (60.2%) were excluded for reasons highlighted in Figure 1, and 45 (39.8%) were included in the scoping review.

### Research Question 1: Study Characteristics

The reviewed body of literature was fairly recent, with the majority of the studies (32/45, 71%) having been published between 2020 and 2022 [45-76]. Most (33/45, 73%) of the articles were from North America and Europe [46,47, 49-55,57,58,61-63,67-70,73-87], of which most (18/33, 55%) were from the United States [46,47,49-52,54,68,73-77, 79-81,84,87]. The greatest number of AI systems were implemented either in hospital-wide settings (6/45, 13%) [50,55,56,65,74,80] or in radiology (6/45, 13%) [53,56,66,68,73,76]. Most (27/45, 60%) of the studies were authored by a multidisciplinary team [46,47,50-55,58, 59,61,62,64,67,69,70,72,74,75,78-80,82,86-89], with clinical and IT or informatics backgrounds being the most common combination (9/27, 33%) [47,50,55,61,70,74,79,87,89]. Among studies with authors from only 1 domain, the most common background was clinical (8/45, 18%) [63,65,66,68,71,73,76,84]. There was a wide range of study designs. Most (24/45, 53%) used a case-study design, including both single-case [46,49,50,52,53,55-57,59,60,66-68,70,74,75,78-83,85,86] or multiple comparative case designs [53,56,78] (Table 2 and Multimedia Appendix 2).

**Table 2:** Overview of articles included in the scoping review (N=45).

Author, year, country; clinical setting	Study aim	Study design
Anand et al [79], 2018, United States; pediatrics	Describe Child Health Improvement through Computer Automation system and methods to represent pediatric guidelines using Arden syntax	Case study
Baxter et al [50], 2020, United States; hospital-wide implementation	Conduct a detailed analysis of barriers to use of machine learning model in health care	Case study
Bennet [77], 2011, United States; mental health	Evaluate the effects of a data-driven clinical productivity system that leverages electronic health record data to provide productivity decision support functionality in a real-world clinical setting	Pre-post study
Champion et al [87], 2011, United States; intensive care	Illuminate barriers and facilitators to use of intensive insulin therapy CDSS^a^	Qualitative study
Chonde et al [68], 2021, United States; radiology	Evaluate the implementation of an AI^b^-powered translation system in radiology	Case study
Chong et al [65], 2021, Australia; hospital-wide implementation	Determine if a VTE^c^ stewardship program can increase risk-appropriate VTE prophylaxis and VTE risk assessment using CDSS	Interrupted time series
Cruz et al [85], 2019, Spain; primary care	Describe a real-time CDSS and its effect on adherence to clinical pathways	Case study
Damoah et al [60], 2021, Ghana; management	Explore how an AI-enhanced medical drone application in Ghana’s health care supply chain improves the health care supply chain system	Case study
Davis et al [73], 2020, United States; radiology	Determine the impact of a machine learning algorithm, meant to mark CT^d^ head examinations pending interpretation as higher probability for intracranial hemorrhage	Case study
Dios et al [83], 2015, Spain; surgery	Present a decision support system for operating room scheduling at a university hospital in Seville, Spain	Case study
García Bermúdez et al [69], 2021, Spain; internal medicine service	Assess the user satisfaction of a virtual caregiver designed to monitor the health of patients admitted to hospital for COVID-19 infection for a period of 30 days after discharge	Quantitative study
Goncalves et al [59], 2020, Brazil; nursing	Present the nurses’ experience with technological tools to support the early identification of sepsis	Case study
Herman et al [64], 2021, Indonesia; public health	Assess the impact of an AI-based application on rifampicin-resistant tuberculosis screening	Qualitative study with key informant interviews
Kalil et al [88], 2018, Brazil; surgery	Describe the impact of a new risk-management cognitive robot related to the processes of identification and care for patients at sepsis risk in a clinical-surgical unit	Retrospective observational study
Kashyap et al [47], 2021, United States; not specified	Identify the different computational and organizational setups that early-adopter health systems have used to integrate an AI-based CDSS into clinical workflows	Qualitative study with key informant interviews
Lacey et al [61], 2020, United Kingdom; surgery	Assess the impact of using automatic video auditing in the quality and quantity of hand-wash events	Interrupted time series
Lai et al [52], 2020, United States; public health	Describe the implementation of a digitally automated prehospital triage solution to direct patients to appropriate care	Case study
Litvin et al [84], 2012, United States; primary care	Describe use of a CDSS on antibiotic prescribing for acute respiratory infections in primary care, as well as facilitators and barriers to adoption	Mixed methods
McKillop et al [48], 2021, multiple regions; public health	Characterize the diverse use cases of COVID-19–related conversational agents built using the IBM Watson Assistant platform	Cross-sectional study
Mohamed et al [71], 2021, United Arab Emirates; dentistry	Validate and implement the AI system and quantify referral patterns to the orthodontist specialist before and after implementation of the system	Quantitative survey
Moorman [49], 2021, United States; inpatient care	Describe the experiences and lessons learned during implementation of AI system	Case study
Morales et al [72], 2021, Brazil; emergency care	Describe early implementation of a digital triage and monitoring service that included the use of a chatbot using algorithmic decision-making	Observational study
Ng et al [45], 2021, Singapore; general care	Develop a predictive model for risk stratification for enrollment into a nationwide transitional care program	Analysis of existing data set
O’Neil et al [76], 2021, United States; radiology	Assess (1) whether the introduction of an algorithm for the detection of intracerebral hemorrhage at noncontrast CT affects turnaround times and (2) whether the impact on turnaround time was dependent on the manner in which information was presented in the radiologist workflow	Quasi-experimental study
Petitgand et al [67], 2020, Canada; emergency department	Analyze the implementation of an AI-based decision support system in an emergency department focusing on actors’ representations of the system	Case study
Rais et al [82], 2018, Portugal; management	Discuss optimization approaches for logistics services in hospitals	Case study
Rath et al [81], 2017, United States; surgery	Describe the development, implementation, and evaluation of a model-based decision support system to determine daily scheduling of anesthesiologists and rooms for elective surgeries	Case study
Reis et al [55], 2020, Germany; hospital-wide implementation	Describe a failed AI project at a large hospital and identify the root causes that led to failure	Case study
Romero-Brufau et al [51], 2020, United States; primary care	To explore attitudes about AI among staff who used AI-based CDSS	Pre-post study
Romero-Brufau et al [54], 2020, United States; general care units	Reduce unplanned hospital readmissions using AI-based CDSS	Controlled study
Saverino et al [62], 2021, Italy; rehabilitation	Describe the role of a digital AI platform in facilitating the implementation of changes in rehabilitation service during the COVID-19 pandemic	Retrospective observational study
Schlicher et al [75], 2021, United States; management	Discuss the implementation of data analytics in AI-enabled mission control at one of the largest health care service providers in Washington state	Case study
Schuh et al [78], 2018, Austria; intensive care, oncology, and nephrology	Outline the technical and clinical aspects of 3 CDSSs integrated into practice at Vienna General Hospital	Case study describing 3 projects
Semenov et al [86], 2016, Russia; laboratory	Present research and development of a decision support system for the patients of a laboratory service	Case study
Sendak et al [46], 2020, United States; emergency department	Describe the steps taken to integrate Sepsis Watch, a sepsis detection and management platform, into routine care delivery at Duke University Hospital in Durham, North Carolina	Case study
Snowdon et al [74], 2020, United States; interdisciplinary	Describe the system implemented, workflow changes, and impact on vulnerable citizens	Case study
Strohm et al [53], 2020, The Netherlands; radiology	Identify barriers and facilitators to the implementation of AI applications in clinical radiology	Case study (multiple)
Sukums et al [89], 2015, Ghana and Tanzania; primary care	Describe health workers’ acceptance and use of the CDSS for maternal care at rural facilities in Ghana and Tanzania and identify factors affecting successful adoption	Mixed methods
Sun [56], 2021, China; hospital-wide implementation	Study how social power among various stakeholders affects IT adoption in health care	Mixed methods
Tamposis et al [70], 2022, Greece; urology	Present design and implementation of a software platform for supporting detection as well as using and processing clinical, bio-chemical, imaging, and histopathologic findings from fusion biopsy	Case study
Tan et al [66], 2021, Singapore; radiology	Describe the use of AI for automatic detection and flagging of CT findings not reported by radiologists to improve patient safety	Case study
Thurso et al [58], 2021, Slovakia; dentistry	Evaluate the clinical impact of an AI upgrade of an existing orthodontic mobile coaching app	Pre-post study
Wen et al [80], 2019, United States; hospital-wide implementation	Present recommendations for developing natural language processing tool sets based on the experience of developing clinical natural language processing at the Mayo Clinic in Rochester, Minnesota	Case study
Wijnhoven [57], 2021, The Netherlands; neonatal care	Theory formalization of grounded insights from a CDSS development case, and by doing this create an organizational learning theoretical foundation for AI development in organizations	Case study
Wong et al [63], 2021, Canada; oncology	Characterize the impact of deep learning–based auto-segmented contour models in the clinical workflow at 2 cancer centers	User feedback survey

^a^CDSS: clinical decision support system.

^b^AI: artificial intelligence.

^c^VTE: venous thromboembolism.

^d^CT: computed tomography.

### Research Question 2: Types and Applications of AI Technology

The most common type of AI application implemented was automation or optimization technology, reported in 71% (32/45) of the implemented systems [45,46,49-51,53-59,62,64,65, 70,71,73,75,77-79,81-84,86-89]. Other technologies implemented included human language technologies, computer vision, and robotics technology (Table 2 and Multimedia Appendices 2 and 3). The most common AI model was a symbolic or knowledge-based model, reported in nearly half (22/45, 49%) of the reviewed studies [48,52-54,57, 59,68-74,77-80,84,85,88], followed by statistical models (9/45, 20%) [45,49-51,58,81,82]. The most commonly performed task was recognition (16/45, 36%) [52,56,61, 63-66,72,73,76,78-80,84,85], followed by forecasting (9/45, 20%) [45,46,49-51,53,54,57,71]. Other tasks performed were event detection, goal-driven optimization, interaction support, and personalization (Table 2, Multimedia Appendices 2 and 3). Although more than half (24/45, 53%) of the AI applications had no action autonomy [46,48-51,53,54,57, 63,66,67,70,73-75,79,81-85,87-89], a few reported applications had low (2/21, 10%) [55,72], medium (4/21, 19%) [58,69,71,86], or high (6/21, 29%) [52,55,60,61,68,76] action autonomy (Table 2, Multimedia Appendices 2 and 3). Nearly three-quarters of all AI systems were intended for clinical care (33/45, 73%) [46,49,51,53-59,61,63-73,78-80,84-89], and the majority (18/33, 55%) of these concerned providing support to inform the patient-provider encounter [46,49,51,55,56,61,63, 65,67,68,74,78,79,84,85,87,89], followed by diagnosis and prediction-based diagnosis (13/33, 39%) [53,55,57,59,64,66,70,71,73,78,80,86,88]. The remaining AI systems (12/45, 27%) were intended for health systems management and planning [45,50,52,60,62,74-77,81-83]. Health care providers were the most common target users; most often physicians (19/45, 42%) [46,49,51,53-55,57-64,66-68, 70,71,73,74,76,79,80,84,85,88,89], followed by nurses (6/45, 13%) [46,49,51,59,87,88]. Other intended users included health workers, technicians, managers, patients or caregivers, and the general public (Table 2 and Multimedia Appendix 2).

### Research Question 3: Implementation Process Characteristics

The research focus in approximately a third of the studies was to present the effectiveness of the implemented intervention (16/45, 36%) [54,58,60-62,65,66,71,73-75,77,81,82,85,88]. Other research foci included user experiences [51,59,63,64,69,86], AI use metrics [48,52,80,84,89], and identification of barriers or facilitators [50,53,55,57,67,87] (Table 2, Multimedia Appendices 2 and 3). Most (32/45, 71%) of the studies described the implementation process as successful, and only a few (4/45, 9%) described it as unsuccessful (in the rest of the studies, the success of the implementation was either not mentioned, or the outcome was inconclusive).

In a little more than half (23/45, 51%) of the reviewed studies, the motives behind the implementation were not described. For those studies that did (22/45, 49%), we identified 6 types of motives, with *Improve health care quality* and *Achieve better patient outcomes* being the 2 most common. Studies in the former theme described AI systems used to improve quality of services [46,71,75,87,88], reduce diagnostic errors [66], reduce hospital length of stay [73], or reduce unplanned readmissions [50,54], whereas studies in the latter theme described AI systems used to achieve better patient survival [59,70]. Another theme, *Improve efficiency*, focused on health care–cost reduction, increased service production, and optimization of public services [45,72,74,76,77]. *Respond to the COVID-19 pandemic* was stated as a motive necessitated by the need for access to the most up-to-date information [48], the sudden surge in demand for health care services [52], prioritization of limited resources [72], and reorganization of service delivery in response to local guidelines for prevention of infection transmission [62]. *Improve provider satisfaction* focused on workload reduction for health care professionals [55,69]. *Empower patients* by using AI to support interpretations of laboratory investigations, rather than just the test results, was another motive for implementing AI [86].

Of the 45 included studies, 3 (7%) had an explicit focus on implementation processes [46,49,68]. In the other studies, characteristics common to implementation processes were identified: cocreation, contextualization, nondisruptive workflow design, communication, learning focus, training, incentives, and organizational strategies. Both barriers and facilitators were described.

Several (8/45, 18%) implementation efforts involved *cocreation* with multidisciplinary stakeholders, starting from an ideation phase that included problem identification, requirement collection, and design or redesign of clinical workflows to facilitate AI-system integration [45,46,49,52,55,59,68,78]. Cocreation also involved end users in the design of user interfaces [46,68]. *Contextualization* of AI systems relating to the local context and target population was highlighted as important in development and implementation [52,54]. *Nondisruptive workflow design* was emphasized, where efforts were made to design AI systems around existing roles and functions of the intended user to avoid radical modification of current practice to fit the AI system [46,49,51]. *Communication* efforts were seen as central to building trust and promoting use by sharing evidence of AI effectiveness with clinicians and describing overall benefits of the technology [46,49,59], appointing champions to promote AI among peers [46,53,75], and encouraging informal communication between clinicians and IT developers to cultivate relationships and build trust in the AI [56]. However, the study by Sendak et al [46] encouraged the separation of developers and clinicians and made conscious efforts to shift focus away from the technical aspects of AI. A *learning focus* could begin in the ideation phase to understand and assess the problem to be addressed by AI before coding, through development and implementation, by iteratively testing and adjusting workflows [46]. After implementation, learning continued through the continuous capture of user feedback to enable improvement [68]. *Training* involved both informal and formal sessions to enable AI use [56,89]. After implementation, training could continue in formal peer-group meetings to share best practices and individual training and support for more reluctant users [84]. *Incentives* were used to promote or enforce AI use. More controlling approaches included periodic monitoring and audits [56,84] or removing alternative ways of performing the task altogether to necessitate AI use [84]. Gamification was used to promote a feeling of reward and competition [61,65]. *Organizational efforts* involved including the hospital’s top leadership as essential members of the project team and the design and implementation of the AI system to promote uptake [49,55]. One organization formed a special governance committee as a formal mechanism to monitor AI use among health care providers [46]. Another organization’s innovation strategy included innovation managers as part of the organizational structure to promote AI [53].

In 7% (3/45) of the studies [50,57,68], the use of the following implementation frameworks was mentioned: the Reach, Effectiveness, Adoption, Implementation, and Maintenance framework [90]; the Nonadoption, Abandonment, Scale-up, Spread, and Sustainability framework [91]; and the Socialization, Externalization, Combination, and Internalization model of knowledge dimensions [92]. Of the 45 included studies, 4 (9%) proposed new frameworks, principles, or recommendations based on their presented findings and implementation experiences [49,55,56,80]. Moorman [49] proposed 6 principles for implementation of AI: elements of trust and transparency, minimal impact on workflows, stakeholder buy-in, relevant education, actionability of AI outputs, and sustainability through follow-up interactions. Reis et al [55] proposed a framework for overcoming cognitive and affective resistance to AI implementation centered around concerns of users (physicians), such as transparency and understandability of the AI system, involvement of users in the AI training, and trust in the AI system. Sun [56] proposed a power strategy matrix for AI adoption, suggesting that a “boss strategy” or “expert strategy” can influence adoption. Wen et al [80] presented 3 desiderata for developing an AI-based platform, where the second one focused on improving adoption.

## Discussion

### Principal Findings

Our aim with this study was to explore how the implementation of AI in health care practice has been empirically investigated in the research literature. We found that research on implementation of AI systems is mostly published in high-income countries, covers many different clinical settings and disciplines, and predominantly focuses on care providers as users. The AI models are primarily symbolic or knowledge based, use automation or optimization technologies, and are mainly used to perform tasks related to recognition. AI systems are predominantly intended for clinical care, particularly clinical care pertaining to patient-provider encounters. Most possess no action autonomy but rather support human decision-making. The focus of most research is on establishing the effectiveness of interventions or related to technical and computational aspects of AI systems. Focus on the specifics of implementation processes does not yet seem to be a priority in research, and the use of frameworks to guide implementation is rare.

### Study Characteristics

Most of the studies were published very recently (2020-2022), which is unsurprising given the temporal distribution of AI health care studies. Research on AI implementation in health care is predominantly conceptual in nature, dominated by commentaries, perspectives, opinion articles, and conceptual frameworks that raise important questions and issues but without much-needed empirical evidence [93-96]. As the empirical evidence base for the implementation of AI solutions in routine health care is still narrow and premature, it limits possibilities for generalization both for practice and for the advancement of methodological approaches. Most of the articles were published in high-income countries, particularly the United States. This finding is consistent with the more developed digital health infrastructure, routine use of electronic health records, and big data initiatives in North American and European countries and aligns with other reviews of AI applications in various fields of health care [32,97,98]. The many different clinical settings and disciplines could corroborate the data-driven nature of health care; the fact that AI is highly applicable; or that because of its nascent state, AI is still being tried in many different contexts. Given the focus on clinical care, it is not surprising that the intended users were mostly health care providers, particularly physicians. A recent scoping review on the use of AI in primary care found a similar predominance of physicians as target end users [99]. This suggests a view of AI systems as tools to support decision-making by physicians rather than other health professionals. It was surprising to find a scarcity of implementations of AI applications to handle infectious diseases (except for the study by McKillop et al [48]), given the overwhelming attention given to, and funding provided for, the management of the COVID-19 pandemic in 2020-2022. Another underrepresented area where AI holds a strong promise is mental health (except for the studies by Bennett [77] and Rahman et al [100]).

### Types and Applications of AI Technology

Nearly half of the AI models were symbolic or knowledge based. They used human-generated logical representations, rules, and ontologies to infer conclusions and have greater explainability than models that are based on pure data-driven or statistical approaches. However, they might not live up to the full potential of AI because they are “hard-coded, expert cookbooks” that are limited by the knowledge that is encoded into them [101]. Data-driven, statistical approaches such as machine learning learn predictive functions based on the inputted data. However, these methods are opaque and have implications for health care in relation to patient or provider trust, accountability and quality assurance, and patient safety [3,102]. The World Health Organization’s guidance on ethics and governance of AI for health recognizes the potential trade-off between transparency and accuracy but encourages AI explainability and transparency over black-box approaches [43]. The predominance of knowledge-based or symbolic models, whose greater transparency and longer existence may ease acceptance among care providers, is in line with previous reviews [103]. However, the majority of recently published AI models use data-driven or hybrid technologies, and knowledge-based models comprised only a minority of the applications [104]. Our study found that automation or optimization technologies were by far the most common, followed by human language technologies. More than half of the AI systems implemented had no action autonomy. Instead, they were human decision support systems where the AI system cannot act on its recommendation or output but depends on the human operating the system to use or disregard the recommendation made by it. This finding indicates that decision support systems are the types of AI systems that have achieved adoption the earliest, likely because they enhance human actions and cause minimal disruption to clinical workflows [105].

### Implementation Process

This study found that the way the implementation process of AI systems in health care is researched is varied and builds on many types of study designs and methodologies. A little more than half of the included studies did not provide a clear motivation for implementing an AI system, which is a key factor for successful adoption of AI in health care [105]. The lack of a clear motivation indicates poor alignment with well-defined needs from clinical practice and risks reinforcing a technology-focused logic regarding implementation of AI in health care. This observation might reflect the lack of consistent understanding of what is meant by implementation of AI in daily practice and a lack of methodological consistency in how such implementations should be researched and reported. Most of the studies either had a technical or computational understanding of implementation or viewed implementation in terms of the effectiveness of the intervention. There was not much focus on the actual process of implementation studies but more on presenting cases of implementation. This indicates the relatively nascent nature of evidence in this field and is similar to other studies, which highlights that many of the publications on AI in health care focus on the methods and technical aspects of applying the AI model to clinical scenarios but provide very little information on the actual process of its implementation in practice [51,99].

Despite the limited focus in the studies on researching the implementation process, our inductive analysis identified the following implementation elements: cocreation, designing nondisruptive workflows, maintaining a learning focus, communication, contextualization, leadership and conducive organizational structure, trainings, and enforcement or incentivization of AI use. These aspects are not unique to AI but have been highlighted as important interventions for the adoption of all digital technologies, including AI; for example, the involvement of end users in the design and implementation of IT services and applications forms the basis of user-centered design, which is seen as an important driver of uptake of digital technologies [106]. The commitment, involvement, and accountability of leaders is also a well-known factor for successful implementation in practice [107]. Seamless integration with existing workflows was another factor highlighted as central to adoption of AI systems. This finding is consistent with the fact that most studied cases of AI system implementation were based on decision support systems that have no action autonomy and can be conveniently incorporated into routine workflows. However, it is challenging to draw generalized conclusions on the AI implementation strategies from such systems because they introduce incremental improvements in the workflows and do not represent more disruptive types of AI systems; for example, those with high action autonomy.

The findings of this study corroborate the recent work by Gama et al [37] regarding the uncertainty of what should be considered AI and the notion that our understanding of implementation is still in the early stages of development. We would add that this understanding is made even more complex by the lack of agreement on what is meant by the term implementation. We rejected numerous studies during the screening because the term implementation was used in a computational sense; for example, the product concept or requirements were *implemented* as a code, or the coded algorithm was *implemented* using an existing data set. Even in studies involving real-world settings, the term was used to mean execution of a plan without reflection on the process of execution. The focus of implementation as an intentional effort designed to change routine practice, adapt interventions, or increase the uptake of interventions into routine practice was scarce in the published literature.

### Limitations and Methodological Considerations

The strengths of this study include the substantial number of records reviewed and the rigor observed during the screening process. The search strategy was comprehensive and broad, and covered 5 different electronic databases. However, we did not include a broader search of the gray literature that would have undoubtedly captured additional cases and potentially identified more cases representing ongoing or completed implementation projects not yet published in the research literature. As we aimed to investigate the experiences from implementation in clinical practice, during screening we removed clinical trials, case reports, pilots, feasibility studies, and other forms of limited and controlled introduction of AI applications in practice. We expect there to be a lag between the work of technology companies and care providers and subsequent academic publications. However, because of the number of records we identified and the previously found extensive availability of opinion-based articles in the literature in the form of perspectives, insights, and narrative reviews [37], we made a conscious choice to focus on peer-reviewed articles. Although this procedure might risk excluding relevant knowledge from smaller or unsuccessful implementation attempts or other research adjacent to implementation processes, we delimited the results to the literature based on actual experiences from implementation in everyday clinical practice.

Our initial screening of title and abstracts did not require decisions by 2 reviewers, but all decisions in the full-text screening were confirmed in pairs. We deliberately worked to maintain consistency and mitigate individual variation through biweekly meetings where we worked to establish a psychologically safe environment that encouraged all authors to raise or flag doubts, discuss the application of exclusion criteria, or consider differing interpretations. When in doubt, we would backtrack or repeat without blame, and all conflicts and uncertainties were resolved through discussion until consensus was reached. Additional meetings were held with other experts in the domain to ensure methodological rigor. Although the Arksey and O’Malley framework for scoping reviews [39] does not include a quality appraisal, we would recommend that future authors consider doing so as the number of articles that carefully consider implementation increases.

### Conclusions

The current body of empirical evidence demonstrates a dissonance between research and practice needs. On the one hand, conceptual and methodological AI research builds on large promises of AI to revolutionize health care and problematizes its slow uptake into practice. On the other hand, the current empirically supported knowledge derives mostly from implementations of AI systems with low action autonomy and highlights lessons on the implementation process that are typical of implementations of other types of information systems. Further research is needed on the more disruptive types of AI systems being implemented in routine care to identify those aspects of implementation unique to AI. This highlights the need for future research to advance in two main streams: (1) to empirically study the implementation processes of various types of AI systems in health care practice and (2) to support empirical research and practical implementations by developing and disseminating an AI-specific implementation framework that would take into account some of the unique aspects related to uptake of AI in health care, such as building trust, addressing transparency issues, developing explainable and interpretable solutions, and addressing ethical concerns around privacy and data protection.

## Appendix

Multimedia Appendix 1 Detailed search strategy for the study.

Multimedia Appendix 2 Overview of articles included in the scoping review (N=45).

Multimedia Appendix 3 Types of artificial intelligence (AI) systems implemented and main focus of research. (A) Types of AI technologies implemented, classified according to the Organisation for Economic Co-operation and Development Framework. (B) Types of tasks performed by AI in health care across the included studies. (C) Level of action autonomy in the AI implemented. (D) Overall focus of the paper and results.

## Abbreviations

AI: artificial intelligence
PRISMA-ScR: Preferred Reporting Items for Systematic Reviews and Meta-Analyses extension for Scoping Reviews
