# Supplementary material for: Artificial Intelligence Applications in Health Care Practice: Scoping Review
Source: J Med Internet Res. 2022 Oct 5;24(10):e40238. doi: 10.2196/40238 (PMC9582911; doi:10.2196/40238)
Supplement: Multimedia Appendix 1 [file jmir_v24i10e40238_app1.docx]

Detailed Search Strategy

1. Pubmed

| **Date of search:** 2021-12-10  **Number of hits:** 2 184  **Comments:** | **Field tags:**  MeSH Terms = Term from the Medline controlled vocabulary, including terms found below this term in the MeSH hierarchy  MeSH Terms:noexp = Does not include terms found below this term in the MeSH hierarchy  Title/Abstract = Words and numbers included in the title, collection title, abstract, and other abstract of a citation  pdat = Date of Publication  " " = Citation Marks, searches for an exact phrase  * = Truncation |
| --- | --- |
| \| artificial intelligence in healthcare \| 4 082 \| ("neural networks, computer"[MeSH Terms:noexp] OR "artificial intelligence"[MeSH Terms:noexp] OR deep learning[MeSH Terms] OR supervised machine learning[MeSH Terms] OR "artificial intelligence"[Title/Abstract] OR deep learning[Title/Abstract] OR "supervised machine learning"[Title/Abstract]) AND ("delivery of health care"[MeSH Terms] OR "health care"[Title/Abstract] OR healthcare[Title/Abstract]) AND (2011:3000[pdat]) AND (english[Filter]) \| \| --- \| --- \| --- \| \| implementation \| 2 535 952 \| (implement*[Title/Abstract] OR improv*[Title/Abstract] OR innovat*[Title/Abstract] OR intervent*[Title/Abstract]) AND (2011:3000[pdat]) AND (english[Filter]) \| \| combined search \| 2 184 \| (implement*[Title/Abstract] OR improv*[Title/Abstract] OR innovat*[Title/Abstract] OR intervent*[Title/Abstract]) AND ("neural networks, computer"[MeSH Terms:noexp] OR "artificial intelligence"[MeSH Terms:noexp] OR deep learning[MeSH Terms] OR supervised machine learning[MeSH Terms] OR "artificial intelligence"[Title/Abstract] OR deep learning[Title/Abstract] OR "supervised machine learning"[Title/Abstract]) AND ("delivery of health care"[MeSH Terms] OR "health care"[Title/Abstract] OR healthcare[Title/Abstract]) AND (2011:3000[pdat]) AND (english[Filter]) \| | |

1. CINAHL (Ebsco)

| **Date of search:** | 2021-12-10 | **Field tags:**  DT = Date of Publication LA = Language  MH = CINAHL Subject Headings  * = Truncation  " " = Citation Marks, searches for an exact phrase |
| --- | --- | --- |
| **Number of hits:** | 826 |  |
| **Comments:** |  |  |
| - Limiters – Peer Reviewed | |  |
| \| artificial intelligence in healthcare \| 1 626 \| (MH "Neural Networks (Computer)" OR MH "Artificial Intelligence" OR MH "Deep Learning" OR MH "Machine Learning" OR "artificial intelligence" OR deep learning OR supervised machine learning) AND ("health care" OR healthcare) AND DT 20110101-20301231 AND LA English \| \| --- \| --- \| --- \| \| implementation \| 859 350 \| (implement* OR improv* OR innovat* OR intervent*) AND DT 20110101-20301231 AND LA English \| \| combined search \| 826 \| ((MH "Neural Networks (Computer)" OR MH "Artificial Intelligence" OR MH "Deep Learning" OR MH "Machine Learning" OR "artificial intelligence" OR deep learning OR supervised machine learning) AND ("health care" OR healthcare)) AND (implement* OR improv* OR innovat* OR intervent*) AND DT 20110101-20301231 AND LA English \| | | |

1. PsycINFO (ProQuest)

| **Date of search:** | 2021-12-10 | **Field tags:** |
| --- | --- | --- |
| **Number of hits:** | 410 | DTYPE = Document type LA = Language  MAINSUBJECT.EXACT = Subject term from APA Thesaurus of Psychological Index Terms  noft = Anywhere except full text  PEER(yes) - retrieves documents that are peer reviewed SCHOL(yes) - retrieves documents that are scholarly YR = Publication year  " " = Citation Marks, searches for an exact phrase  * = Truncation |
| **Comments:** |  |  |
| \| artificial intelligence in healthcare \| 886 \| (MAINSUBJECT.EXACT("Neural networks") OR MAINSUBJECT.EXACT("Artificial intelligence") OR MAINSUBJECT.EXACT("Deep learning") OR MAINSUBJECT.EXACT("Machine learning") OR noft("artificial intelligence" OR deep learning OR supervised machine learning)) AND noft("health care" OR healthcare) AND YR(>=2011) AND DTYPE("article") AND SCHOL(yes) AND  PEER(yes) AND LA(english) \| \| --- \| --- \| --- \| \| implementation \| 439 504 \| noft(implement* OR improv* OR innovat* OR intervent*) AND YR(>=2011) AND DTYPE("article") AND SCHOL(yes) AND  PEER(yes) AND LA(english) \| \| combined search \| 410 \| ((MAINSUBJECT.EXACT("Neural networks") OR MAINSUBJECT.EXACT("Artificial intelligence") OR MAINSUBJECT.EXACT("Deep learning") OR MAINSUBJECT.EXACT("Machine learning") OR noft("artificial intelligence" OR deep learning OR supervised machine learning)) AND noft("health care" OR healthcare)) AND noft(implement* OR improv* OR innovat* OR intervent*) AND YR(>=2011) AND DTYPE("article") AND SCHOL(yes) AND  PEER(yes) AND LA(english) \| | | |

1. Web of Science Core Collection

| **Date of search:** | 2022-01-27 | **Field tags:** |
| --- | --- | --- |
| **Number of hits:** | 2 674 | DT= Document Type LA= Language  PY= Year published  TS= Topic  Searches for topic terms in the following fields within a record. Title  Abstract  Author Keywords Keywords Plus®  " " = Citation Marks, searches for an exact phrase  * = Truncation |
| **Comments:**  Editions: SCI expanded, SSCI, ESCI | | DT Article  "Article: Reports of research on new and original works that are considered citable. Includes research papers, brief communications, technical notes, chronologies, full papers, and case reports (presented like full papers) that were published in a journal and/or presented at a symposium or conference."  (Not including "Reviews, Review of Literature, Mini-reviews, and Systematic reviews. If an article is listed under the review section in a journal and/or Review of Literature appears in the title it will be assigned a review.") |
| \| artificial intelligence in healthcare \| 5 329 \| TS=(artificial intelligence OR deep learning OR neural networks OR supervised machine learning) AND TS=(health care OR healthcare) AND PY=(2011-2030) AND LA=(English)  AND DT=(Article) \| \| --- \| --- \| --- \| \| implementation \| 3 887 282 \| TS=(implement* OR improv* OR innavat* OR intervent*) AND PY=(2011-2030) AND LA=(English) AND DT=(Article) \| \| combined search \| 2 674 \| TS=(artificial intelligence OR deep learning OR neural networks OR supervised machine learning) AND TS=(health care OR healthcare) AND PY=(2011-2030) AND TS=(implement* OR improv* OR innavat* OR intervent*) AND PY=(2011-2030) AND LA=(English) AND DT=(Article) \| | | |

1. Scopus

| **Date of search:** 2022-02-16  **Number of hits:** 3 085  **Comments:** | **Field tags:**  TITLE-ABS-KEY = A combined field that searches abstracts, keywords, and document titles.  PUBYEAR = year of publication  DOCTYPE ar = documents classified as articles  " " = Citation Marks, searches for a loose phrase  * = Truncation |
| --- | --- |
| \| artificial intelligence in healthcare \| 6 455 \| TITLE-ABS-KEY("artificial intelligence" OR "deep learning" OR "neural networks" OR "supervised machine learning") AND TITLE-ABS-KEY("health care" OR healthcare) AND PUBYEAR  > 2010 AND DOCTYPE(ar) AND LANGUAGE(english) \| \| --- \| --- \| --- \| \| implementation \| 4 383 909 \| TITLE-ABS-KEY(implement* OR improv* OR innavat* OR intervent*) AND PUBYEAR > 2010 AND DOCTYPE(ar) AND  LANGUAGE(english) \| \| combined search \| 3 085 \| (TITLE-ABS-KEY("artificial intelligence" OR "deep learning" OR "neural networks" OR "supervised machine learning") AND TITLE-ABS-KEY("health care" OR healthcare)) AND TITLE- ABS-KEY(implement* OR improv* OR innavat* OR intervent*) AND PUBYEAR > 2010 AND DOCTYPE(ar) AND  LANGUAGE(english) \| | |
