# Supplementary material for: Artificial Intelligence Applications in Health Care Practice: Scoping Review
Source: J Med Internet Res. 2022 Oct 5;24(10):e40238. doi: 10.2196/40238 (PMC9582911; doi:10.2196/40238)
Supplement: Multimedia Appendix 2 [file jmir_v24i10e40238_app2.docx]

**Overview of articles included in the scoping review (N=45).**

| **Author** | **AI^b^ technology; AI model** | **Task performed; levels of autonomy** | **Intended use of AI; intended user of AI** | **Research focus** | **Motives for implementation; elements in the implementation process** |
| --- | --- | --- | --- | --- | --- |
| Anand et al [79] | Automation or optimization; symbolic model | Recognition; no action autonomy | Clinical care, patient-provider relationship; physicians | Computational performance or technical development | Not mentioned; not mentioned |
| Baxter et al [50] | Automation or optimization; statistical model | Forecasting; no action autonomy | Health care systems, management and planning; unclear | Identification of barriers or facilitators | Health care quality; not mentioned |
| Bennet [77] | Automation or optimization; symbolic model | Goal-driven optimization; unclear | Health care systems, management and planning; unclear | Intervention effectiveness | Efficiency; not mentioned |
| Champion et al [87] | Automation or optimization; statistical model | Goal-driven optimization; no action autonomy | Clinical care, patient-provider relationship; nurses | Identification of barriers or facilitators | Health care quality; not mentioned |
| Chonde et al [68] | Human language technologies; symbolic model | Interaction support; high action autonomy | Clinical care, patient-provider relationship; technicians | Implementation process | Not mentioned; cocreation and learning focus |
| Chong et al [65] | Automation or optimization; unclear | Recognition; unclear | Clinical care, patient-provider relationship; unclear | Intervention effectiveness | Not mentioned; incentivization |
| Cruz et al [85] | Human language technologies; symbolic model | Recognition; no action autonomy | Clinical care, patient-provider relationship; physicians | Intervention effectiveness | Not mentioned; not mentioned |
| Damoah et al [60] | Robotics; unclear | Goal-driven optimization; high action autonomy | Health care systems, management and planning; health workers | Intervention effectiveness | Not mentioned; not mentioned |
| Davis et al [73] | Automation or optimization; symbolic or knowledge based | Recognition; no action autonomy | Clinical care, diagnosis, and prediction-based diagnosis; physicians | Intervention effectiveness | Health care quality; not mentioned |
| Dios et al [83] | Automation or optimization; statistical model | Goal-driven optimization; no action autonomy | Health care systems, management and planning; health managers | Computational performance or technical development | Not mentioned; not mentioned |
| García Bermúdez et al [69] | Human language technologies; symbolic model | Interaction support; medium action autonomy | Clinical care, shifting to home-based care; patients or caregivers | User experience and acceptance | Provider satisfaction; not mentioned |
| Goncalves et al [59] | Automation or optimization; symbolic model | Event detection; unclear | Clinical care, diagnosis, and prediction-based diagnosis; nurses | User experience | Health care quality; cocreation and communication |
| Herman et al [64] | Automation or optimization; unclear | Recognition; unclear | Clinical care, diagnosis, and prediction-based diagnosis; health workers | User experience | Not mentioned; not mentioned |
| Kalil et al [88] | Automation or optimization; symbolic model | Event detection; no action autonomy | Clinical care, diagnosis, and prediction-based diagnosis; nurses | Intervention effectiveness | Health care quality; not mentioned |
| Kashyap et al [47] | Not specified; not specified | Not specified; not specified | Not specified; not specified | Organizational structure | Not mentioned; not mentioned |
| Lacey et al [61] | Computer vision; unclear | Recognition; high action autonomy | Clinical care, patient-provider relationship; physicians | Intervention effectiveness | Not mentioned; incentivization |
| Lai et al [52] | Human language technologies; symbolic model | Recognition; high action autonomy | Health care systems, management and planning; general public | AI use metrics | COVID-19 pandemic; cocreation and contextualization |
| Litvin et al [84] | Automation or optimization; symbolic model | Recognition; no action autonomy | Clinical care, patient-provider relationship; physicians | AI use metrics | Not mentioned; training and incentivization |
| McKillop et al [48] | Human language technologies; symbolic model | Interaction support; no action autonomy | Public health and public health surveillance; general public | AI use metrics | COVID-19 pandemic; not mentioned |
| Mohamed et al [71] | Automation or optimization; symbolic model | Forecasting; medium action autonomy | Clinical care, diagnosis, and prediction-based diagnosis; physicians | Intervention effectiveness | Health care quality; not mentioned |
| Moorman [49] | Automation or optimization; statistical model | Forecasting; no action autonomy | Clinical care, patient-provider relationship; physicians, nurses, and technicians | Implementation process | Not mentioned; cocreation, nondisruptive workflows, communication, and organizational structure |
| Morales et al [72] | Human language technologies; symbolic model | Recognition; low action autonomy | Clinical care, patient-provider relationship; patients or caregivers | AI use metrics | Efficiency and COVID-19 pandemic; not mentioned |
| Ng et al [45] | Automation or optimization; statistical model | Forecasting; unclear | Health care systems, management and planning; unclear | Computational performance or technical development | Efficiency; cocreation |
| O’Neil et al [76] | Computer vision; unclear | Recognition; high action autonomy | Health care systems, management and planning; physicians | Intervention effectiveness | Efficiency; not mentioned |
| Petitgand et al [67] | Human language technologies; unclear | Event detection; no action autonomy | Clinical care, patient-provider relationship; physicians | Identification of barriers or facilitators | Not mentioned; not mentioned |
| Rais et al [82] | Automation or optimization; statistical model | Goal-driven optimization; no action autonomy | Health care systems, management and planning; unclear | Intervention effectiveness | Not mentioned; not mentioned |
| Rath et al [81] | Automation or optimization; statistical model | Goal-driven optimization; no action autonomy | Health care systems, management and planning; health managers | Intervention effectiveness | Not mentioned; not mentioned |
| Reis et al [55] | Automation, optimization, and human language technologies; hybrid model | Event detection and interaction support; low action autonomy (physician use), high action autonomy (patient use) | Clinical care, patient-provider relationship; physicians, patients, or caregivers | Identification of barriers or facilitators | Provider satisfaction; cocreation and organizational structure |
| Romero-Brufau et al [51] | Automation or optimization; statistical model | Forecasting; no action autonomy | Clinical care, patient-provider relationship; physicians and nurses | User experience | Not mentioned; nondisruptive workflows |
| Romero-Brufau et al [54] | Automation or optimization; symbolic model | Forecasting; no action autonomy | Clinical care, patient-provider relationship; physicians | Intervention effectiveness | Health care quality; contextualization |
| Saverino et al [62] | Automation or optimization; hybrid model | Goal-driven optimization; unclear | Health care systems, management and planning; health workers | Intervention effectiveness | COVID-19 pandemic; not mentioned |
| Schlicher et al [75] | Automation or optimization; unclear | Goal-driven optimization; no action autonomy | Health care systems, management and planning; unclear | Intervention effectiveness | Health care quality; communication |
| Schuh et al [78] | Automation or optimization; symbolic model | Recognition and event detection; unclear | Clinical care, patient-provider relationship, diagnosis, and prediction-based diagnosis; unclear | Computational performance or technical development | Not mentioned; cocreation |
| Semenov et al [86] | Automation or optimization; unclear | Event detection; medium action autonomy | Clinical care, diagnosis, and prediction-based diagnosis; patients or caregivers | User experience | Empower patients; not mentioned |
| Sendak et al [46] | Automation or optimization; hybrid model | Forecasting; no action autonomy | Clinical care, patient-provider relationship; nurses | Implementation process | Health care quality; nondisruptive workflows, communication, learning focus, and organizational structure |
| Snowdon et al [74] | Human language technologies; symbolic model | Personalization; no action autonomy | Health care systems, management and planning; health workers | Intervention effectiveness | Efficiency; not mentioned |
| Strohm et al [53] | Automation or optimization; symbolic model | Forecasting; no action autonomy | Clinical care, diagnosis, and prediction-based diagnosis; physicians | Identification of barriers or facilitators | Not mentioned; communication and organizational structure |
| Sukums et al [89] | Automation or optimization; unclear | Unclear; no action autonomy | Clinical care, patient-provider relationship; physicians | AI use metrics | Not mentioned; training |
| Sun [56] | Automation or optimization, human language technologies, and computer vision; unclear | Recognition, event detection, and interaction support; unclear | Clinical care, patient-provider relationship, diagnosis, and prediction-based diagnosis; physicians, patients, or caregivers | Applying social power lens to adoption of AI | Not mentioned; communication, training, and incentivization |
| Tamposis et al [70] | Automation or optimization; symbolic model | Event detection; no action autonomy | Clinical care, diagnosis, and prediction-based diagnosis; physicians | Computational performance or technical development | Health care quality; not mentioned |
| Tan et al [66] | Computer vision; unclear | Recognition; no action autonomy | Clinical care, diagnosis, and prediction-based diagnosis; physicians | Intervention effectiveness | Health care quality; not mentioned |
| Thurso et al [58] | Automation or optimization; statistical model | Personalization; medium action autonomy | Clinical care, patient role in clinical care; physicians, patients, or caregivers | Intervention effectiveness | Not mentioned; not mentioned |
| Wen et al [80] | Human language technologies; symbolic model | Recognition; unclear | Clinical care, diagnosis, and prediction-based diagnosis; physicians | AI use metrics | Not mentioned; not mentioned |
| Wijnhoven [57] | Automation or optimization; symbolic model | Forecasting; no action autonomy | Clinical care, diagnosis, and prediction-based diagnosis; physicians | Identification of barriers or facilitators | Not mentioned; not mentioned |
| Wong et al [63] | Computer vision; unclear | Recognition; no action autonomy | Clinical care, patient-provider relationship; technicians | User experience | Not mentioned; not mentioned |

^a^RQ: research question.

^b^AI: artificial intelligence.

^c^CDSS: clinical decision support system.

^d^VTE: venous thromboembolism.

^e^CT: computed tomography.
