# Supplementary figures and images for: Artificial Intelligence Applications in Health Care Practice: Scoping Review
Source: J Med Internet Res. 2022 Oct 5;24(10):e40238. doi: 10.2196/40238 (PMC9582911; doi:10.2196/40238)

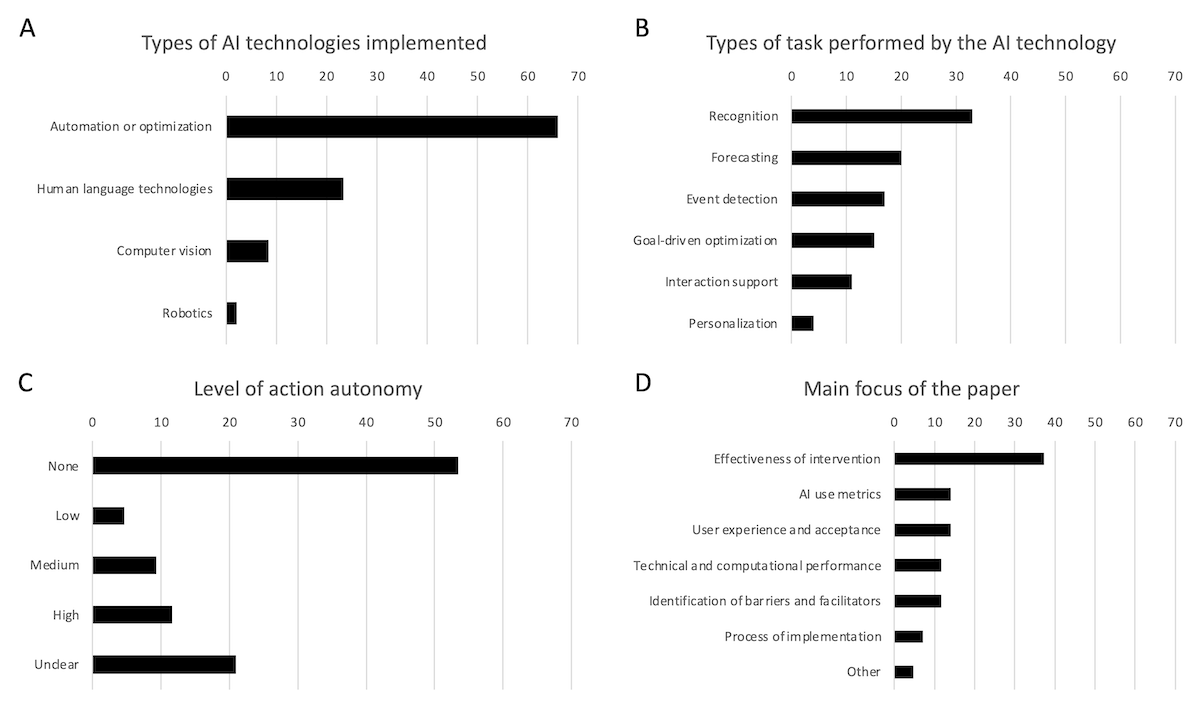

Supplement: Multimedia Appendix 3 [file jmir_v24i10e40238_app3.png]
